# Supplementary material for: Enhanced Photovoltaic Performance in D-π-A Copolymers Containing Triisopropylsilylethynyl-Substituted Dithienobenzodithiophene by Modulating the Electron-Deficient Units
Source: Polymers (Basel). 2018 Dec 21;11(1):12. doi: 10.3390/polym11010012 (PMC6401703; doi:10.3390/polym11010012)
Supplement: Supplementary file 1 [file polymers-11-00012-s001.pdf]

Supplementary Material

Enhanced Photovoltaic Performance in D-π-A Copolymers Containing Triisopropylsilylethynyl-substituted Dithienobenzodithiophene by Modulating the Electron-deficient Units

Junfeng Tong*, Lili An, Jie Lv, Pengzhi Guo, Xunchang Wang, Chunyan Yang, and Yangjun Xia*

1. EXPERIMENTAL SECTION

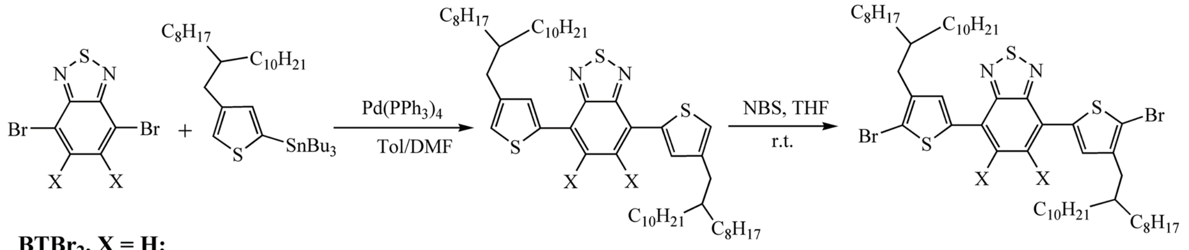

$BTBr_2$, X = H;
$FBTBr_2$, X = F.

DTBT-OD, X = H;
DTFBT-OD, X = F.

DTBT-$ODBr_2$, X = H;
DTFBT-$ODBr_2$, X = F.

Scheme S1 Synthesis of dibromide DTBT-$ODBr_2$ and DTFBT-$ODBr_2$

Synthesis of dibromide DTBT-$ODBr_2$ and DTFBT-$ODBr_2$

4,7-Bis(4-(2-octyldodecyl)thien-2-yl)benzo[*c*][1,2,5]thiadiazole (DTBT-OD)

In a 50 mL two-neck flask, 20 mg $Pd(PPh_3)_4$ (0.017 mmol) was added to a stirring 20 mL toluene and 3 mL *N*,*N*-dimethylformamide (DMF) mixture solution of $BTBr_2$ (0.30 g, 1.02 mmol) and 4-(2-octyldodecyl)-2-tributyltinthiophene (2.16 g, 3.30 mmol). The mixture was stirred at 105 °C for 18 h under the Ar atmosphere. Then cooling to room temperature, the solvent was removed under reduced pressure, and extracted by chloroform (CF) and washed with water and brine. After drying by anhydrous Na_2SO_4, the solvent was removed at reduced pressure. The crude product was purified by chromatography (silica gel, 200–300 mesh) using petroleum (PE) as the eluent to afford 0.77 g red solid. Yield: 88%. M.p.: 57~59 °C. ^1H NMR ($CDCl_3$, 400 MHz), δ (ppm): 7.84 (s, 2H), 7.83 (s, 2H), 7.01 (s, 2H), 2.62 (d, J = 6.8 Hz, 4H), 1.70 (br, 2H), 1.42−1.24 (m, 64H), 0.87 (t, J = 6.8 Hz, 12H). Anal. Calcd for $C_{54}H_{86}Br_2N_2S_3$: C, 63.63%; H, 8.50%; N, 2.75%. Found, C, 63.41%; H, 8.31%; N, 2.85%.

4,7-Bis(5-bromo-4-(2-octyldodecyl)thien-2-yl)benzo[*c*][1,2,5]thiadiazole (DTBT-$ODBr_2$)

Into a 25 mL THF solution of DTBT-OD (0.77 g, 0.89 mmol), *N*-bromosuccinimide (NBS, 0.36 g, 2.05 mmol) was added slowly and stirred at room temperature for 6 h under dark. The react mixture was poured into 100 mL water and extracted with $CHCl_3$, then washed with brine and

water 3 times, respectively. The combined organic layers were dried over anhydrous Na_2SO_4, and the solvents were removed by rotary evaporation. Finally, the crude compound was purified by column chromatography (silica gel 200–300 mesh; eluent: PE) and isolated as a deep-red solid of 0.70 g. Yield: 77%. M.p.: 48~50 °C. ^{1}H NMR ($CDCl_3$, 600 MHz), δ (ppm): 8.09 (s, 2H), 7.83 (s, 2H), 7.18 (s, 2H), 2.65 (d, J = 7.2 Hz, 4H), 1.70 (br, 2H), 1.32−1.20 (m, 64H), 0.87 (t, J = 4.8 Hz, 12H). Anal. Calcd for $C_{54}H_{86}Br_2N_2S_3$: C, 63.63%; H, 8.50%; N, 2.75%. Found, C, 63.43%; H, 8.31%; N, 2.85%.

4,7-Bis(4-(2-octyldodecyl)thien-2-yl)-5,6-difluorobenzo[*c*][1,2,5]thiadiazole (DTFBT-OD)

The coupling procedure of DTFBT was similar to the synthesis of DTBT-OD, except that reagents was replaced by $FBTBr_2$ (0.50 g, 1.51 mmol) and 4-(2-octyldodecyl)-2- tributyltinthiophene (3.47 g, 5.30 mmol). The target compound was obtained as yellow solid of 1.11 g. Yield 82%. M.p.: 61~64 °C. ^{1}H NMR ($CDCl_3$, 400 MHz), δ (ppm): 7.75 (s, 2H), 7.74 (s, 2H), 2.57 (d, J = 7.2 Hz, 4H), 1.75 (br, 2H), 1.32−1.20 (m, 64H), 0.86 (m, 12H). Anal. Calcd for $C_{54}H_{86}F_2N_2S_3$: C, 72.27%; H, 9.66%; N, 3.12%. Found, C, 72.10%; H, 9.51%; N, 3.40%.

4,7-Bis(5-bromo-4-(2-octyldodecyl)thien-2-yl)-5,6-difluorobenzo[*c*][1,2,5]thiadiazole (DTFBT-ODBr$_2$)

The bromination procedure of DTFBTBr$_2$ was similar to the synthesis of DTBT-ODBr$_2$, except that reagents were replaced by DTFBT-OD (0.50 g, 0.56 mmol) and NBS (0.22 g, 1.23 mmol). The target compound was obtained as orange-red solid of 0.51 g. Yield 78%. M.p.: 50~52 °C. ^{1}H NMR ($CDCl_3$, 400 MHz), δ (ppm): 7.94 (s, 2H), 2.62 (d, J = 6.8 Hz, 4H), 1.75 (br, 2H), 1.32−1.26 (m, 64H), 0.86 (t, J = 6.8 Hz, 12H). Anal. Calcd for $C_{54}H_{84}F_2N_2S_3$: C, 61.46%; H, 8.02%; N, 2.65%. Found, C, 61.21%; H, 7.91%; N, 2.70%.

Synthesis of bistin 2,7-bis(trimethyltin)-5,10-bis(triisopropylsilylethynyl)dithieno[2,3-*d*:2′,3′-*d*′]-benzo[1,2-*b*:4,5-*b*′]dithiophene (DTBDT-TIPSSn)[S1]

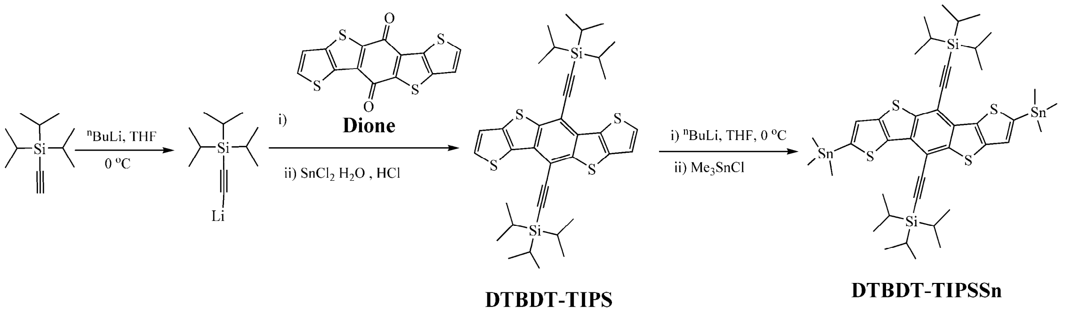

Scheme S2 Synthesis of bistin comonomer DTBDT-TIPSSn

5,10-Bis[triisopropylsilylethynyl]dithieno[2,3-*d*:2′,3′-*d*′]benzo[1,2-*b*:4,5-*b*′]dithiophene (DTBDT-TIPS)

To a solution of trimethylsilylacetylene (2.64 g, 14.45 mmol) in 120 mL anhydrous THF, 5.5 mL ^{n}BuLi (2.5 M, 14.45 mmol) was added dropwise at 0 °C. Then 2.00 g (6.02 mmol) of dithieno[2,3-*d*:2′,3′-*d*′]benzo[1,2-*b*:4,5-*b*′]dithiophene-5,10-diketone suspended in 20 mL THF was added in one portion. The mixture was refluxed overnight under Ar. $SnCl_2{\cdot}2H_2O$ (5.70 g, 25.3 mmol) and hydrochloric acid (10%, 6 mL) were added into the solution. After the reaction was carried out for another 2 h, the cooled solution was poured into 200 mL distilled water, and extracted with CH_2Cl_2 three times. The combined organic phase was dried with anhydrous Na_2SO_4. The solvent was removed under reduced pressure to afford a yellow solid. The resulting yellow solid was purified by chromatography to give a yellow needle crystalline solid (1.80 g, yield, 65%). M.p.: 218–220 °C. ^{1}H NMR ($CDCl_3$, 400 MHz), δ (ppm): 7.63 (d, J = 6.4 Hz, 2H), 7.39 (d, J = 6.4 Hz, 2H), 1.27 (s, 42H). Anal. Calcd for $C_{36}H_{46}S_4Si_2$: C, 65.20%; H, 6.99%. Found, C, 65.21%;

H, 7.05%.

DTBDT-TIPSSn

DTBDT-TIPS (1.05 g, 1.59 mmol) was dissolved in 50 mL anhydrous THF and cooled in an ice bath under Ar atmosphere. 3.30 mL ^{n}BuLi solution (2.5 M in hexane) was added dropwise, and the solution was kept in the ice bath for 0.5 h and at ambient temperature for 0.5 h under vigorous stirring. The mixture was cooled in an ice bath again and trimethyltin chloride (1.21 g, 6.04 mmol) in dry *n*-hexane solution was added and kept in the ice bath for another 0.5 h. Then the solution was stirred at ambient temperature overnight. The mixture was quenched with 5 mL water and extracted with hexane. The organic extract was dried with anhydrous Na_2SO_4 and the solvent was removed under reduced pressure. The resultant solid was recrystallized in iso-propanol 4 times to yield DTBDT-TIPSSn as colorless needles (0.8 g, yield, 53.69%). M.p.: 212–213 ºC. ^{1}H NMR ($CDCl_3$, 400 MHz), δ (ppm): 7.40 (t, J = 11.6 Hz, 2H), 1.28 (s, 42H), 0.44 (t, J = 28 Hz, 18H). Anal. calcd for: $C_{42}H_{62}S_4Si_2Sn_2$: C, 51.02; H, 6.32. Found: C, 51.58; H, 6.48%.

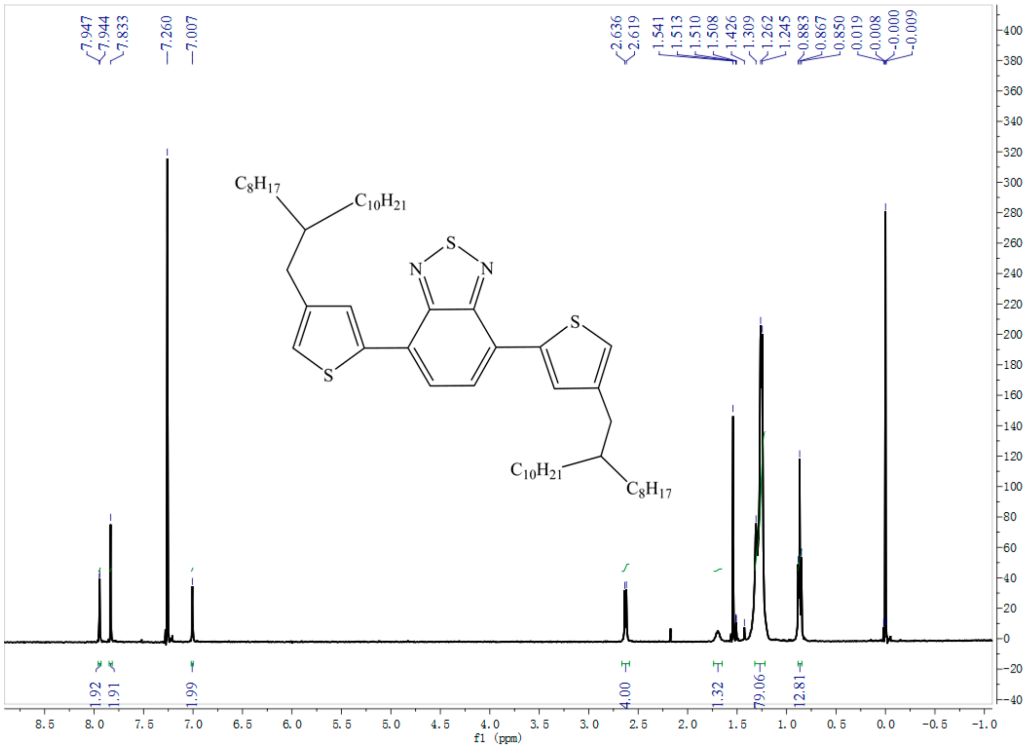

Fig. S1 ^{1}H NMR spectrum of DTBT-OD in $CDCl_3$

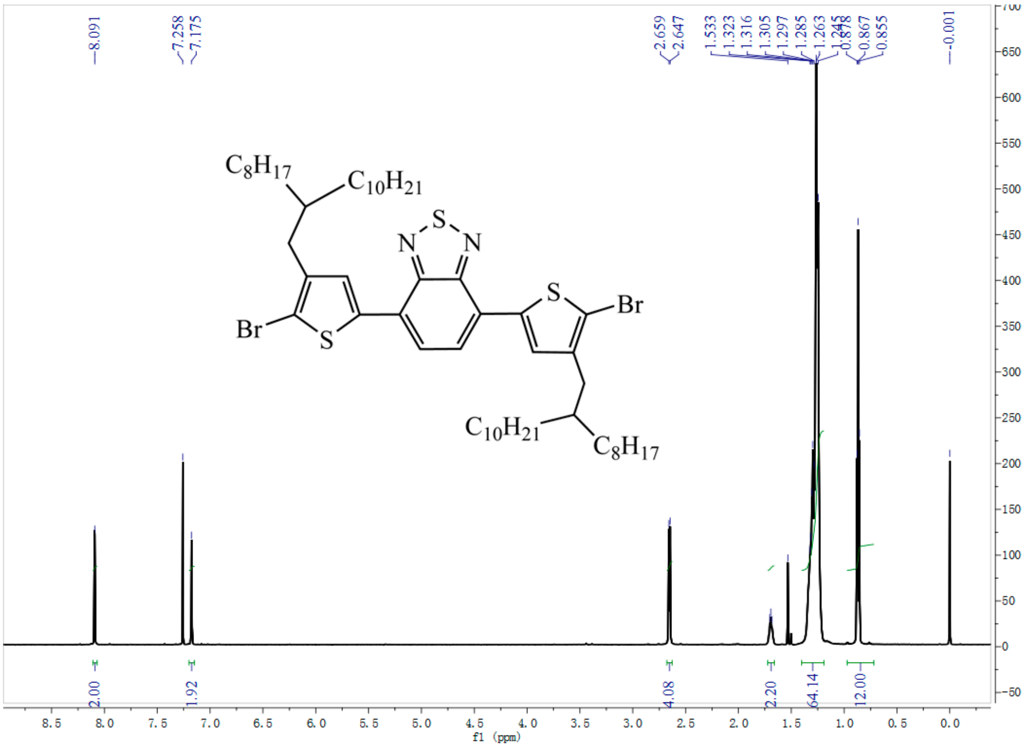

Fig. S2 ^{1}H NMR spectrum of DTBT-ODBr$_2$ in CDCl$_3$

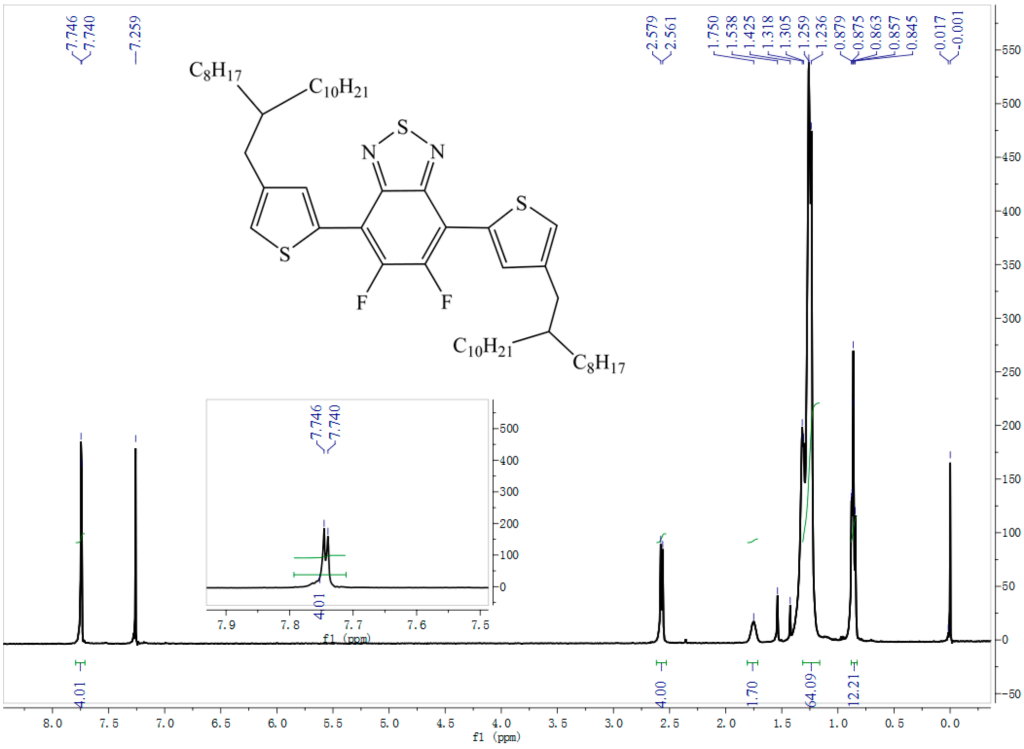

Fig. S3 ^{1}H NMR spectrum of DTFBT-OD in CDCl$_3$

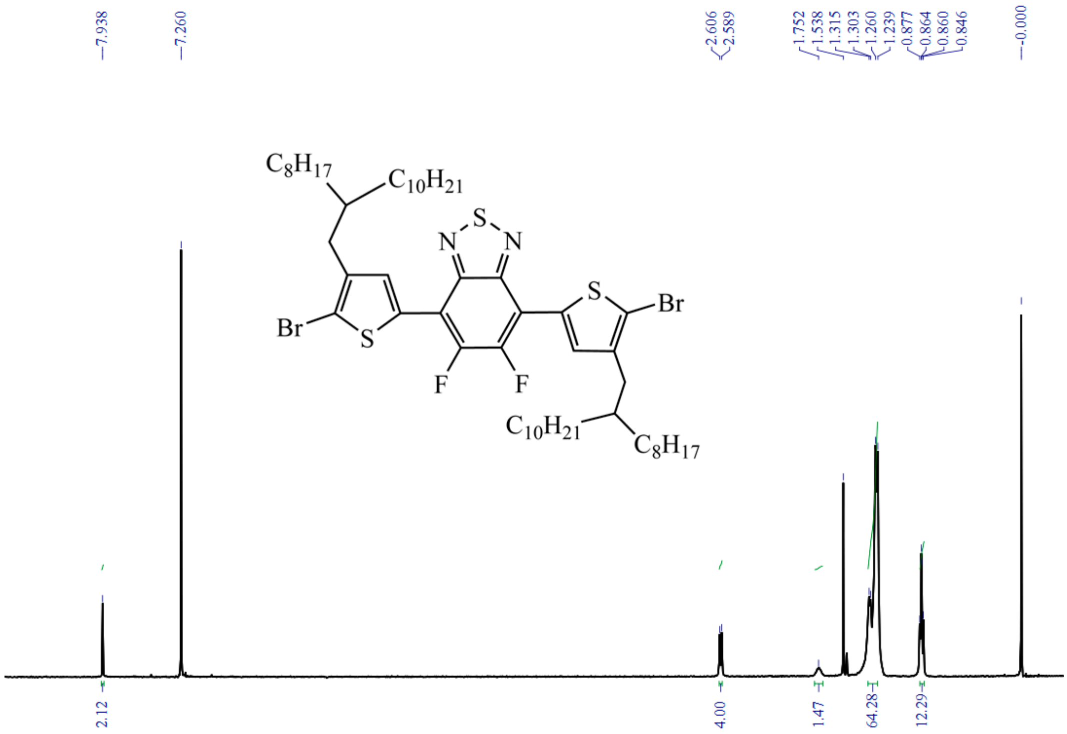

Fig. S4 ^{1}H NMR spectrum of DTFBT-ODBr$_2$ in CDCl$_3$

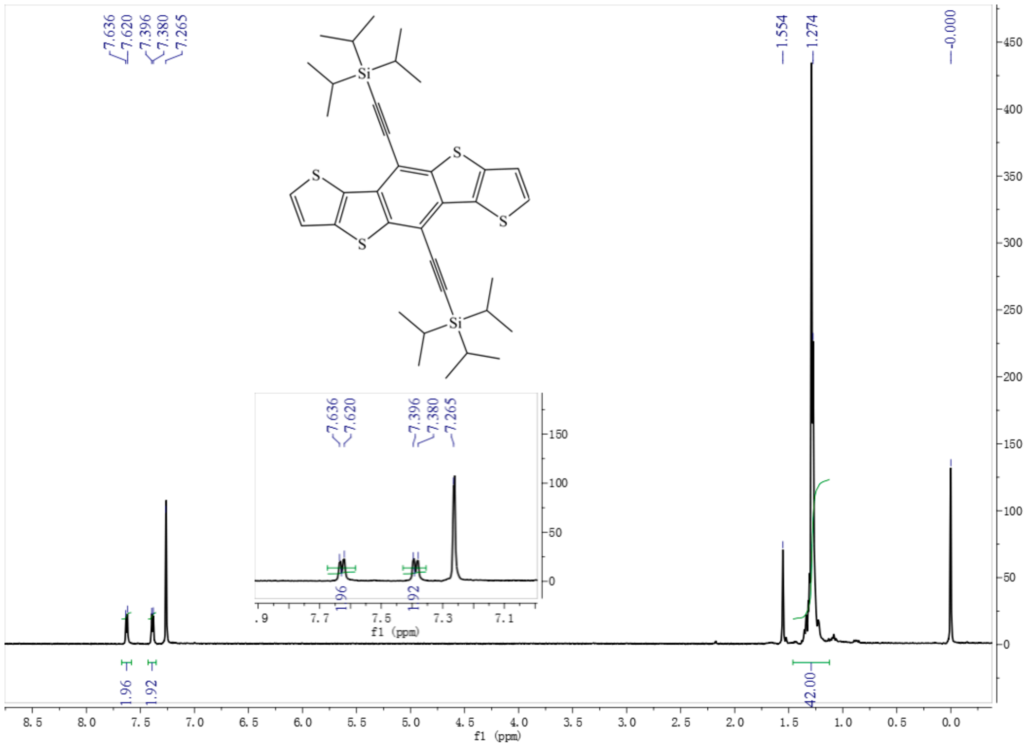

Fig. S5 ^{1}H NMR spectrum of DTBDT-TIPS in CDCl$_3$

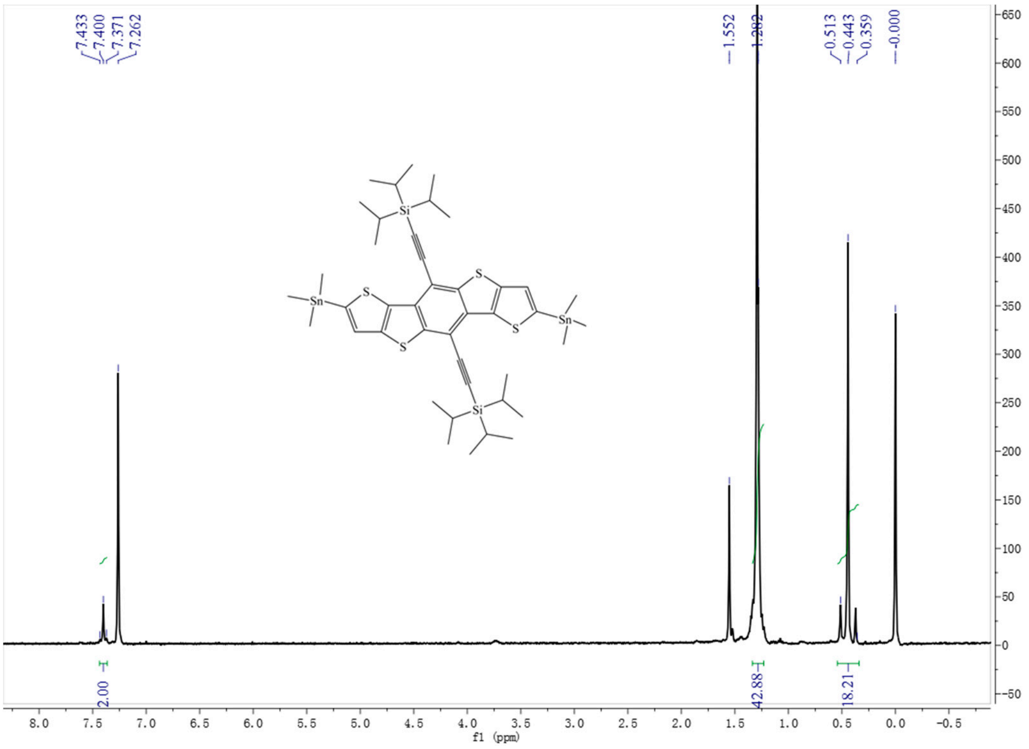

Fig. S6 ^{1}H NMR spectrum of DTBDT-TIPSSn in $CDCl_3$

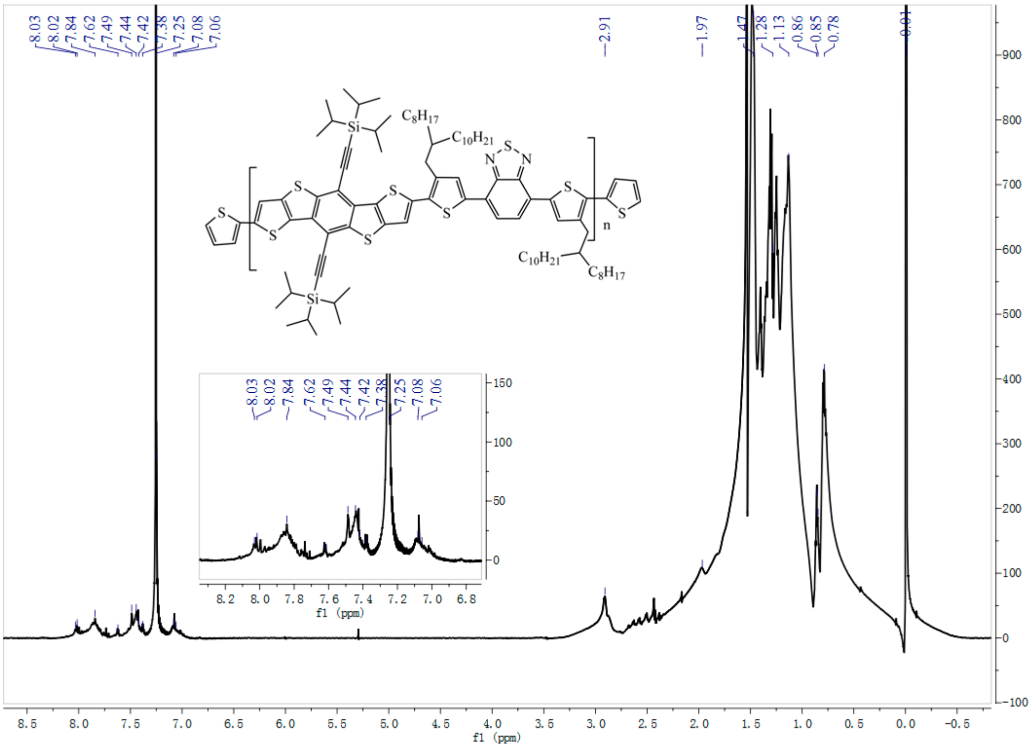

Fig. S7 ^{1}H NMR spectrum of PDTBDT-TIPS-DTBT-OD in $CDCl_3$

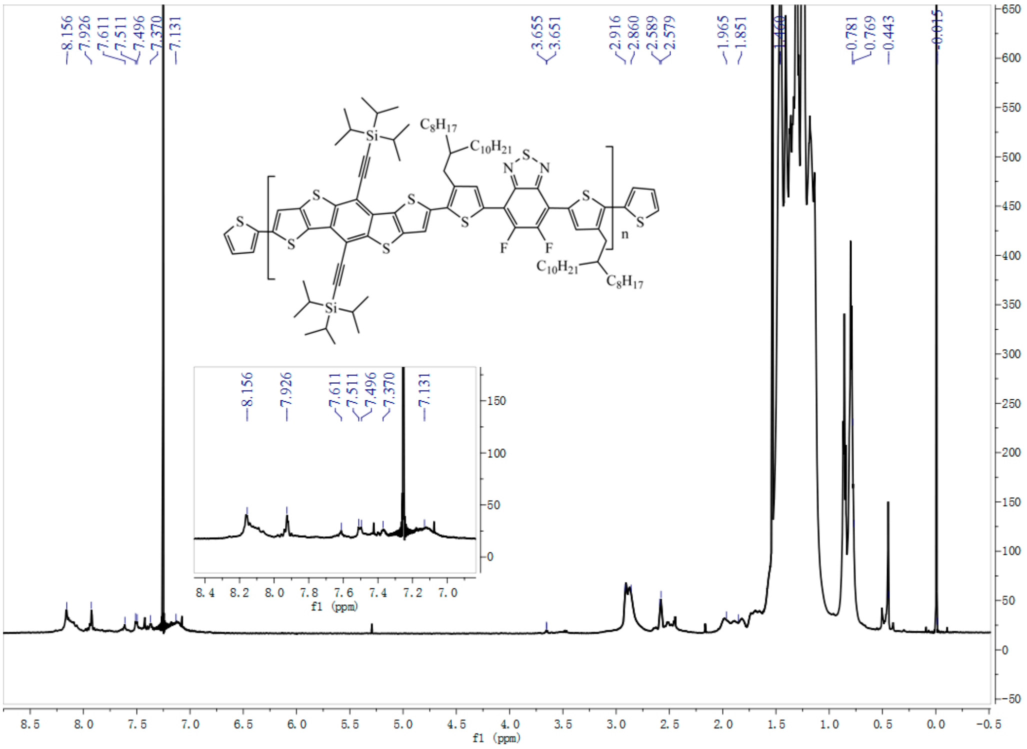

Fig. S8 ^{1}H NMR spectrum of PDTBDT-TIPS-DTFBT-OD in $CDCl_3$

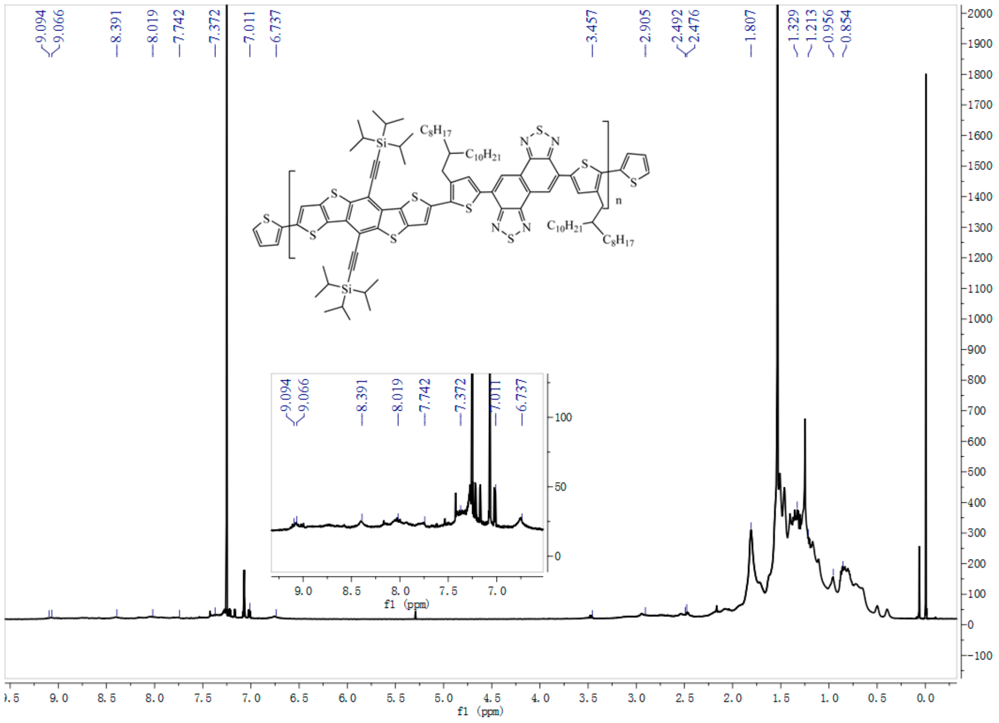

Fig. S9 ^{1}H NMR spectrum of PDTBDT-TIPS-DTNT-OD in $CDCl_3$.

2. RESULTS AND DISCUSSION SECTION

Table S1 Yields, GPC data, thermal properties for DTBDT-TIPS-based polymers.

Polymer	Yield (%)	M_n (kDa)	M_w (kDa)	PDI	T_d (°C)
PDTBDT-TIPS-DTBT-OD	65.5	13.4	24.1	1.80	387
PDTBDT-TIPS-DTFBT-OD	56.9	14.4	27.4	1.90	352
PDTBDT-TIPS-DTNT-OD	81.2	13.6	26.4	1.94	365

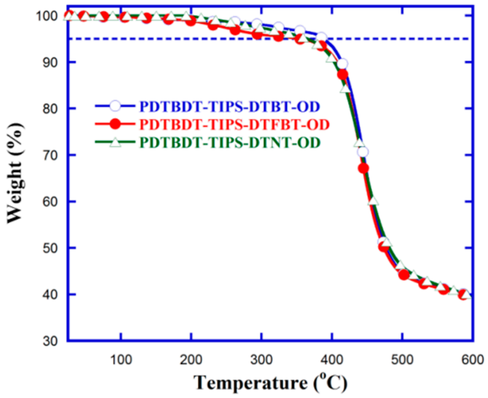

Fig. S10 The TGA curves of copolymers containing DTBDT-TIPS.

Table S2 Absorption coefficients for copolymers.

Polymer	ε_{soln} (L mol^{-1} cm^{-1})	ε_{film} (cm^{-1})
PDTBDT-TIPS-DTBT-OD	59660 (λ = 552 nm)	34129 (λ = 582 nm)
PDTBDT-TIPS-DTFBT-OD	40943 (λ = 535 nm)	30354 (λ = 582 nm)
PDTBDT-TIPS-DTNT-OD	57788 (λ = 632 nm)	35899 (λ = 635 nm)

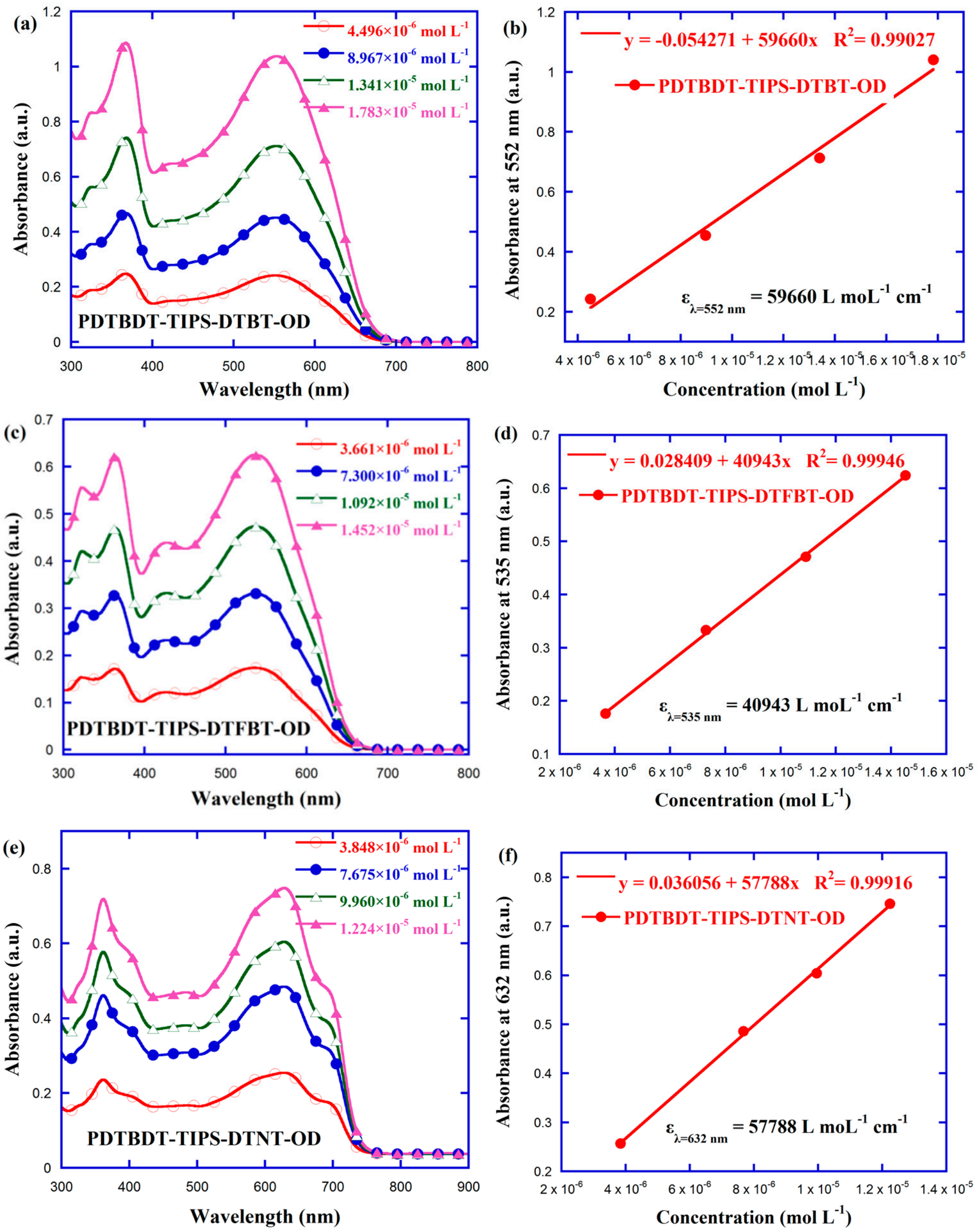

Fig. S11 UV-vis absorption spectra of DTBDT-TIPS-based copolymers dissolved in CB at various concentrations and calculation of molar absorption coefficient.

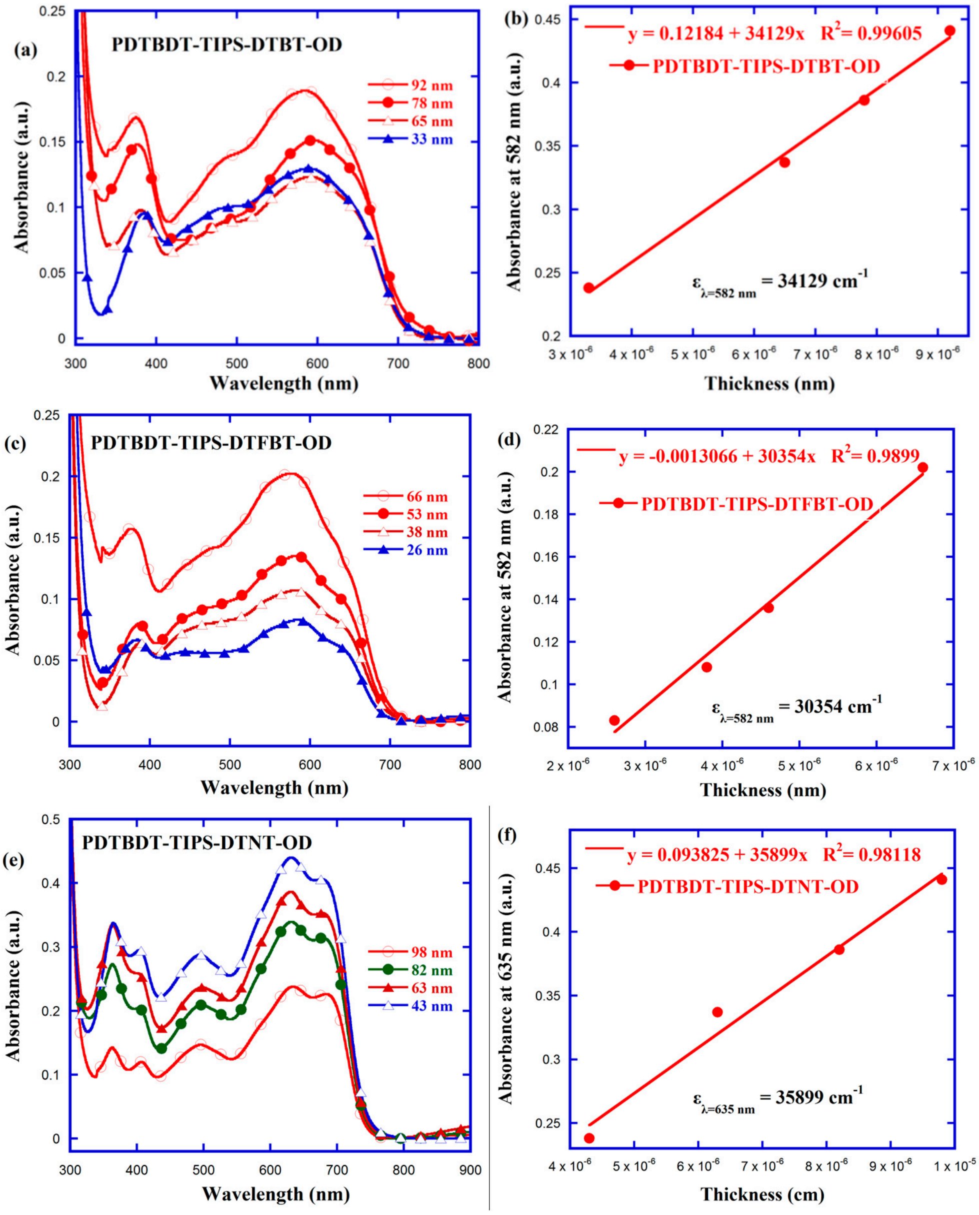

Fig. S12 UV-vis absorption spectra of DTBDT-TIPS-based copolymers in film at varied thickness and calculation of molar absorption coefficient.

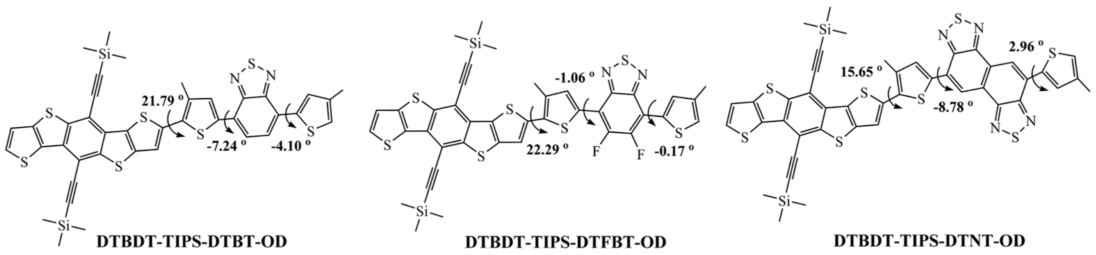

Fig. S13 Dihedral angles for the unimer model compound of copolymers.

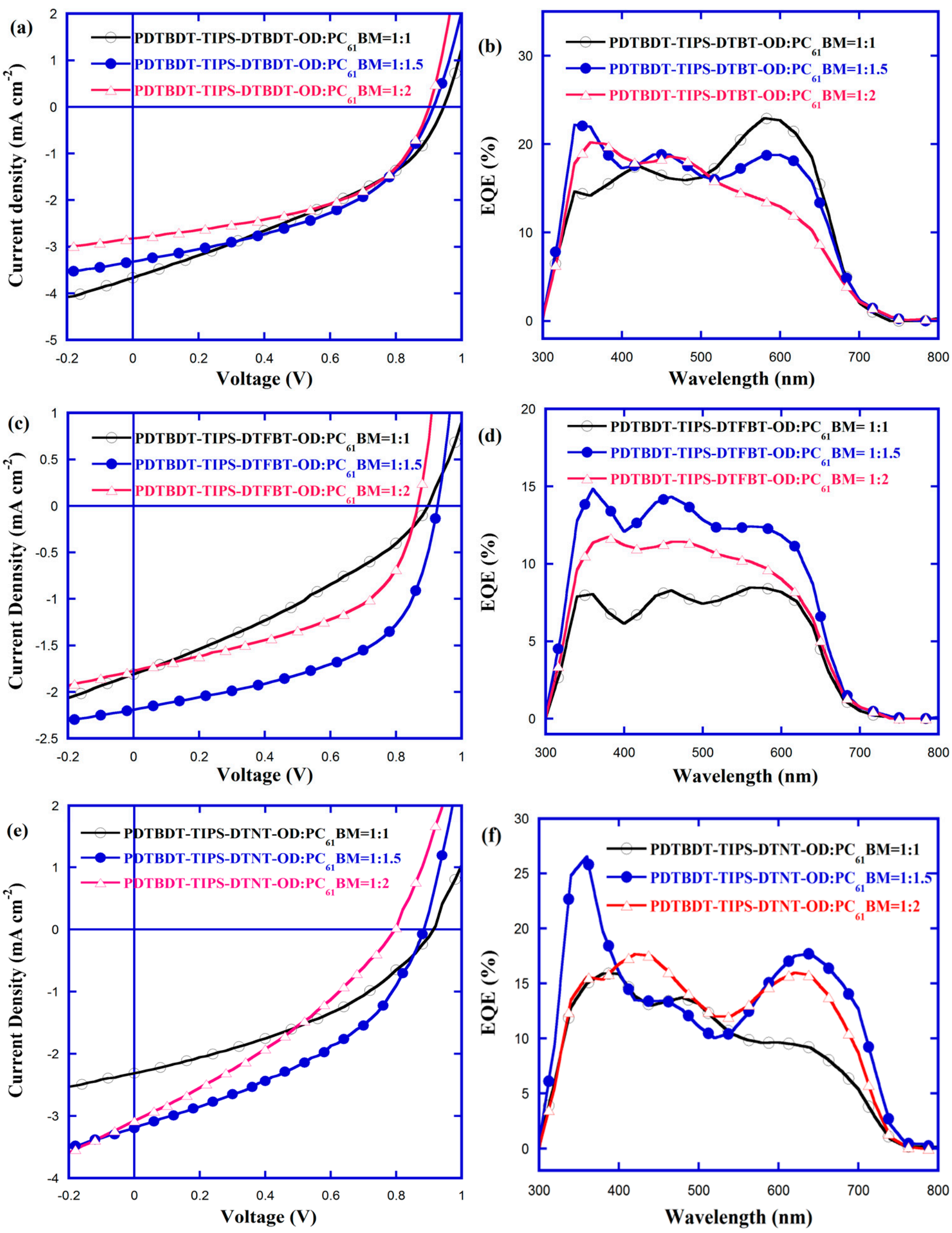

Fig. S14 The *J-V* curves of DTBDT-TIPS-based polymers with different D/A weight ratio to $PC_{61}BM$ and EQE spectra of corresponding PSCs.

Table S3. The photovoltaic performance of the PSCs devices with different blend ratio, without/with 3%(volume) DIO, and using $PC_{71}BM$ replacing of $PC_{61}BM$.

Active layer (w:w)	DIO	V_{OC} (V)	J_{SC} (mA/cm^2)	*FF* (%)	PCE (%)
**PDTBDT-TIPS-DTBT-OD:**$PC_{61}BM$ (1:1)	0%	0.95	3.63	36.49	1.26
**PDTBDT-TIPS-DTBT-OD:**$PC_{61}BM$ (1:1.5)	0%	0.91	3.32	45.21	1.37
**PDTBDT-TIPS-DTBT-OD:**$PC_{61}BM$ (1:2)	0%	0.90	2.82	50.04	1.27
**PDTBDT-TIPS-DTBT-OD:**$PC_{61}BM$ (1:1.5)	3%	0.96	1.89	56.93	1.03
**PDTBDT-TIPS-DTBT-OD:**$PC_{71}BM$ (1:1.5)	0%	0.80	3.62	50.96	1.47
**PDTBDT-TIPS-DTFBT-OD:**$PC_{61}BM$ (1:1)	0%	0.90	1.81	32.58	0.53
**PDTBDT-TIPS-DTFBT-OD:**$PC_{61}BM$ (1:1.5)	0%	0.93	2.19	53.59	1.09
**PDTBDT-TIPS-DTFBT-OD:**$PC_{61}BM$ (1:2)	0%	0.87	1.78	48.48	0.75
**PDTBDT-TIPS-DTFBT-OD:**$PC_{61}BM$ (1:1.5)	3%	0.90	1.03	39.58	0.44
**PDTBDT-TIPS-DTFBT-OD:**$PC_{71}BM$ (1:1.5)	0%	0.86	2.79	45.53	1.07
**PDTBDT-TIPS-DTNT-OD:**$PC_{61}BM$ (1:1)	0%	0.91	2.31	38.15	0.87
**PDTBDT-TIPS-DTNT-OD:**$PC_{61}BM$ (1:1.5)	0%	0.88	3.19	40.55	1.15
**PDTBDT-TIPS-DTNT-OD:**$PC_{61}BM$ (1:2)	0%	0.80	3.08	32.12	0.79
**PDTBDT-TIPS-DTNT-OD:**$PC_{61}BM$ (1:1.5)	3%	0.87	7.03	49.39	3.03
**PDTBDT-TIPS-DTNT-OD:**$PC_{71}BM$ (1:1.5)	3%	0.88	7.21	52.99	3.37

Table S4 Hole mobilities of polymers and blend measured by SCLC model.

Active layer	Ratios/Additive	Thickness (nm)	Slope	μ_h (cm^2 V^{-1} s^{-1})
**PDTBDT-TIPS-DTBT-OD:$PC_{71}BM$**	1:1.5/0%DIO	87	10.26	2.32×10^{-5}
**PDTBDT-TIPS-DTFBT-OD:$PC_{61}BM$**	1:1.5/0%DIO	80	15.23	3.98×10^{-5}
**PDTBDT-TIPS-DTNT-OD:$PC_{71}BM$**	1:1.5/3%DIO	127	11.51	9.09×10^{-5}

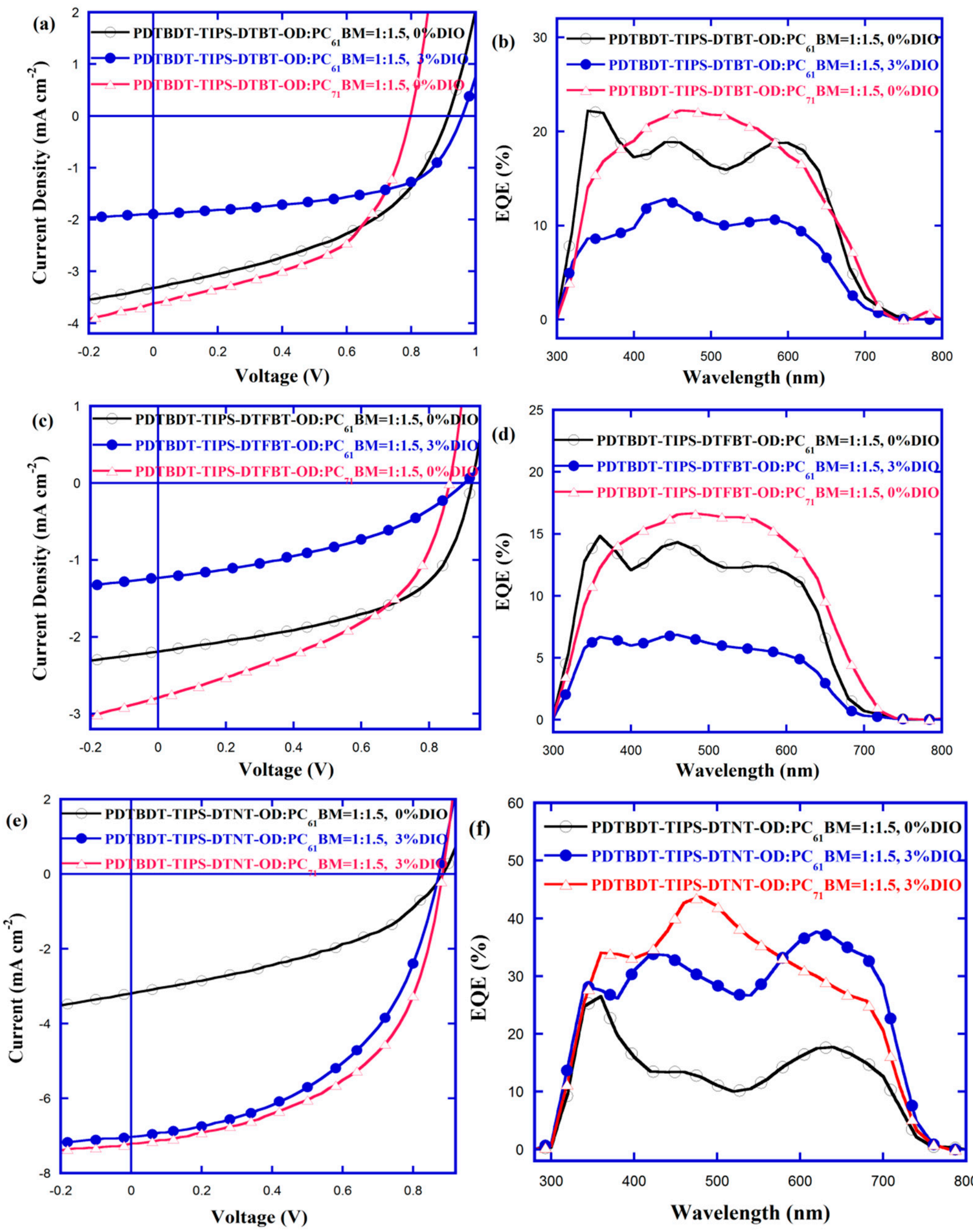

Fig. S15 The *J-V* curves of polymers without and with 3%DIO, $PC_{61}BM$ replacing with $PC_{71}BM$ and EQE spectra of corresponding PSCs.

Reference

[S1] S. Sun, P. Zhang, J. Li, Y. Li, J. Wang, S. Zhang, Y. Xia, X. Meng, D. Fan, J. Chu, *J. Mater. Chem. A* **2014**, *2*, 15316–15325.
